# Plant responses to *Agrobacterium tumefaciens* and crown gall development

**DOI:** 10.3389/fpls.2014.00155

**Published:** 2014-04-23

**Authors:** Jochen Gohlke, Rosalia Deeken

**Affiliations:** ^1^School of Plant Sciences, University of ArizonaTucson, AZ, USA; ^2^Department of Molecular Plant Physiology and Biophysics, Julius-von-Sachs-Institute, University of WuerzburgWuerzburg, Germany

**Keywords:** plant defenses, phytohormones, morphological adaptions, metabolomic changes, epigenetics

## Abstract

*Agrobacterium tumefaciens* causes crown gall disease on various plant species by introducing its T-DNA into the genome. Therefore, *Agrobacterium* has been extensively studied both as a pathogen and an important biotechnological tool. The infection process involves the transfer of T-DNA and virulence proteins into the plant cell. At that time the gene expression patterns of host plants differ depending on the *Agrobacterium* strain, plant species and cell-type used. Later on, integration of the T-DNA into the plant host genome, expression of the encoded oncogenes, and increase in phytohormone levels induce a fundamental reprogramming of the transformed cells. This results in their proliferation and finally formation of plant tumors. The process of reprogramming is accompanied by altered gene expression, morphology and metabolism. In addition to changes in the transcriptome and metabolome, further genome-wide (“omic”) approaches have recently deepened our understanding of the genetic and epigenetic basis of crown gall tumor formation. This review summarizes the current knowledge about plant responses in the course of tumor development. Special emphasis is placed on the connection between epigenetic, transcriptomic, metabolomic, and morphological changes in the developing tumor. These changes not only result in abnormally proliferating host cells with a heterotrophic and transport-dependent metabolism, but also cause differentiation and serve as mechanisms to balance pathogen defense and adapt to abiotic stress conditions, thereby allowing the coexistence of the crown gall and host plant.

## INTRODUCTION

*Agrobacterium tumefaciens* causes crown gall disease on a wide range of host species by transferring and integrating a part of its own DNA, the T-DNA, into the plant genome (Chilton et al., 1977). This unique mode of action has also made the bacterium an important tool in plant breeding. After attachment of *Agrobacterium* to plant cells and expression of multiple virulence (vir) genes, several effector proteins, together with T-DNA, are transported into the plant cell by a type-IV-secretion system (Thompson et al., 1988; Ward et al., 1988, 2002; Kuldau et al., 1990; Shirasu et al., 1990; Beijersbergen et al., 1994). Plant factors assist with T-DNA integration into the plant genome (Gelvin, 2000; Mysore et al., 2000; Tzfira et al., 2004; Magori and Citovsky, 2012). After integration, expression of the T-DNA-encoded oncogenes iaaH, iaaM, and ipt induces biosynthesis of auxin and cytokinin (Morris, 1986; Binns and Costantino, 1998). Increased levels of these phytohormones result in enhanced proliferation and formation of crown galls. Despite the transfer of bacterial proteins into the plant cell, most *Agrobacterium* strains do not elicit a hypersensitive response (HR), which is associated with rapid and localized death of cells (Staskawicz et al., 1995). Such a response often occurs when plants are challenged by bacterial pathogens and serves to restrict the growth and spread of pathogens to other parts of the plant. Accordingly, no systemic, broad-spectrum resistance response throughout the plant (systemic acquired resistance, SAR) is induced. Within the first several hours of co-cultivation, pathogen defense response pathways are activated more or less strongly depending on the plant system and *Agrobacterium* genotype used for infection (Ditt et al., 2001, 2006; Veena et al., 2003; Lee et al., 2009). Defense responses become stronger during crown gall development. Furthermore, the physiological behavior of the transformed cells changes drastically. In contrast to the articles which focus on the molecular mechanism utilized by the bacterium to transform the plant cell, here we review the latest findings on the responses of the host plant and in the crown gall to *Agrobacterium* infection. Special attention is paid to the role of gene expression regulation, phytohormones, and metabolism.

## HOST RESPONSES TO *Agrobacterium tumefaciens* BEFORE T-DNA Transfer

### PATHOGEN DEFENSE

The recognition of microbial pathogens plays a central role in the induction of active defense responses in plants. The conserved flagellin peptide flg22 is recognized by the receptor kinase FLS2 and induces the expression of numerous defense-related genes to trigger resistance to pathogenic bacteria (Gómez-Gómez et al., 1999, 2001; Zipfel et al., 2004; Chinchilla et al., 2006). However, the genus *Agrobacterium* fails to induce this type of rapid and general defense response because of an exceptional divergence in the N-terminal conserved domain of flagellin (Felix et al., 1999). When comparing early gene expression changes after infection with the virulent *Agrobacterium* strain C58 with application of the bacterial peptide elf26 (after 1 and 3 h, respectively), dampening of host responses becomes apparent with *Agrobacterium* treatment. The elf26 peptide, a highly conserved motif of one of the most abundant proteins in microbes recognized by the receptor kinase EFR, is a fragment of the elongation factor Tu (EF-Tu). EF-Tu triggers innate immunity responses associated with disease resistance in *Arabidopsis* (Kunze et al., 2004). While treatment with pure elf26 induces gene expression changes of 948 *Arabidopsis* genes (Zipfel et al., 2006), only 35 genes are induced after infection with the virulent *Agrobacterium* strain C58, suggesting that the bacterium somehow neutralizes the response to elf26 by the host plant (Lee et al., 2009). It should be mentioned that the *Arabidopsis* ecotype and age (seedling vs. adult stalk) used in the studies may also account for some of the differences in defense response.

Concerning the transcriptional activation of genes involved in early plant defense responses, several studies have come to different conclusions. *Ageratum conyzoides* cell cultures showed differential expression of defense genes as early as 24 h post infection with a non-oncogenic hypervirulent *Agrobacterium* strain (Ditt et al., 2001). In tobacco suspension cultures infected with different *Agrobacterium* strains, transcription of defense genes increased within 3–6 h, but started to decrease with the onset of T-DNA-transfer (Veena et al., 2003). A study using suspension-cultured cells of *Arabidopsis* did not show changes in transcript levels within 4 to 24 h but activation of defense genes 48 h after infection (Ditt et al., 2006). When agrobacteria are inoculated at the base of wounded *Arabidopsis* stems just very few defense genes are activated 3 h post infection compared to uninfected wounded stems (Lee et al., 2009). In contrast to cell cultures, the latter experimental setup does neither require phytohormone pre-treatment nor virulence gene induction prior to infection. Phytohormone pre-treatment of the cell culture systems of the earlier studies may alter host cell defense responses. Thus, discrepancies between these studies probably result from the different plant inoculation systems used. Nevertheless, agrobacteria can abuse host defense responses for T-DNA delivery. The mitogen-activated protein kinase MPK3 phosphorylates the *Arabidopsis* VIP1 protein, inducing VIP1 relocalization from the cytoplasm to the nucleus. Nuclear localization of VIP1 increases T-DNA transfer and transformation efficiency (Djamei et al., 2007).

### PHYTOHORMONES

**FIGURE 1:**
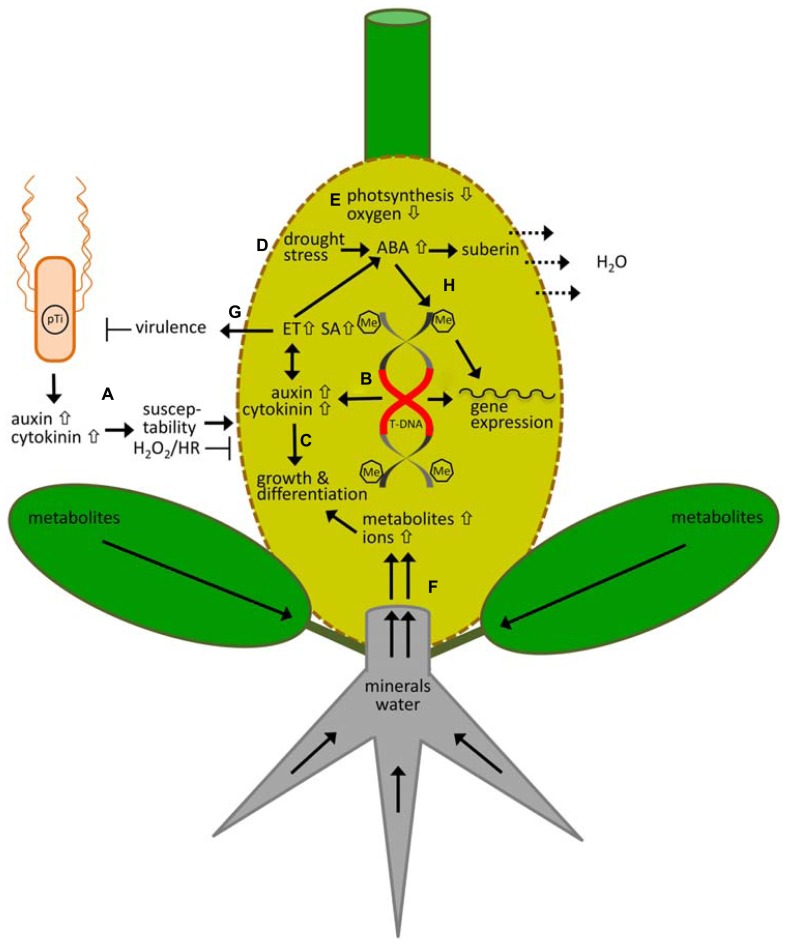
**Responses of the model plant *Arabidopsis thaliana* to *Agrobacterium tumefaciens* and crown gall development**. **(A)** Virulent (pTi) agrobacteria cells themselves produce and release cytokinin and auxin, which increase host susceptibility and inhibit hydrogen peroxide production (H_2_O_2_) and hypersensitive response (HR) at initiation of infection. **(B)** After integration of the bacterial T-DNA into the plant genome, cytokinin and auxin is synthesized by T-DNA encoded enzymes and accumulate inside the tumor. **(C)** This causes massive changes in the gene expression pattern, resulting in metabolomic and morphological adaptations that are necessary for tumor growth and differentiation. **(D)** Loss of water is minimized by drought stress protecting mechanism, which causes an increase in the levels of the stress hormone ABA, and ABA-dependent suberization of cells to prevent water loss. Evaporation of water (H_2_O) from the disrupted crown gall surface drives the flow of water and minerals into crown galls. **(E)** Because photosynthesis is down-regulate the oxygen levels are low, the tumor produces C and N compounds heterotrophically and gains energy mainly anaerobically by alcoholic fermentation. **(F)** Consequently the developing tumor becomes a metabolic sink for the host plant, which accumulates metabolites produced by source leaves and minerals taken up by the roots. **(G)** Auxin and cytokinin also cause an increase in ethylen (ET) which together with salicylic acid (SA) inhibits agrobacterial virulence. **(H)** ABA also induces DNA methylation of the plant genome, thereby regulating gene expression of drought-stress responsive genes. Overall, the crown gall genome becomes hypermethylated (Me) after *Agrobacterium* infection and possibly contributes to the strong changes in gene expression during tumor growth. The oncogenes of the T-DNA remain unaffected by methylation of the plant genome.

Agrobacteria produce auxin and cytokinin themselves in order to modulate plant responses (**Figure 1A**). These phytohormones have been determined in the cells as well as cultivation medium (Morris, 1986). It was postulated that biosynthesis of the phytohormones is catalyzed by enzymes of the T-DNA encoded oncogenes, as transcripts and proteins of these genes were detected in agrobacterial cells (Schröder et al., 1983; Janssens et al., 1984). Pronounced amounts of auxin have been determined in the virulent *Agrobacterium* strain C58 and at lower levels also in plasmidless and T-DNA depleted strains (Liu and Kado, 1979; Kutáèek and Rovenská, 1991). More recent data have confirmed the latter results (Lee et al., 2009). The finding that a strain without a Ti-plasmid still can make auxin implies localization of genes also outside of the Ti-plasmid. However, this assumption is not supported by sequencing data for strain C58 (Wood et al., 2001). Genes known to be involved in auxin biosynthesis seem to be encoded only by the T-DNA of the Ti-plasmid. Recently, these authors determined the presence of iaaH and iaaM transcripts by PCR in *Agrobacterium* cells of strain C58 and confirmed the earlier findings. It remains to be proven whether these genes are responsible for auxin production or if auxin is synthesized by a different mechanism in *Agrobacterium* cells. The mechanism for cytokinin biosynthesis by agrobacteria is far better understood. In nopaline utilizing *Agrobacterium* strains cytokinin is produced in high amounts by the Ti-plasmid encoded *trans*-zeatin synthesizing (tzs) enzyme of which the gene is located in the vir regulon (Akiyoshi et al., 1985, 1987; Hwang et al., 2010). A substantial smaller source for cytokinin production is isopentenylated transfer RNA (tRNA) catalyzed by the chromosomal-encoded enzyme tRNA:isopentenyltransferase (MiaA) present in all *Agrobacterium* strains (Gray et al., 1996).

Earlier studies have shown that pre-treatment of explants with either auxin alone or both auxin and cytokinin increase T-DNA transfer efficiency and stable transformation (Krens et al., 1996; Chateau et al., 2000) as well as crown gall growth (Gafni et al., 1995). In this respect, *Agrobacterium* produced phytohormones play a role at very early time points of infection (**Figure 1A**), before T-DNA-encoded enzymes catalyze synthesis of cytokinin and auxin in the transformed host cell. Concerning the mechanism causing an increase in susceptibility it was speculated that phytohormones induce plant cell division and that the cell cycle phase influences agrobacterial attachment and stable transformation. It seems likely that phytohormone-mediated modification of the physiological state of the cell increases competence for T-DNA transformation and integration. More recent investigations addressed the question about the molecular mechanism and the signaling pathways by which these phytohormones influence host cell susceptibility. Transcriptome microarray data from 3 h after inoculation of *Agrobacterium* strain C58 into *Arabidopsis* stems revealed that the genes known to be involved in phytohormone-dependent signaling are not induced in host cells at this very early time point of infection before transfer of the T-DNA (Lee et al., 2009). It has been shown that indole-3-acetic acid (IAA) has an impact on agrobacterial virulence by inhibiting vir gene induction and growth of agrobacteria (Liu and Nester, 2006). However, this effect was observed with relatively high concentrations of auxin (25–250 μM). In *Agrobacterium* cells the total (free and conjugated) IAA content is 0.3 ± 0.1 μM and in *Arabidopsis* stems 3 h after inoculation with strain C58 it is 2.1 ± 1 μM, whereas in *Arabidopsis* crown galls the content is ca. 10 times higher (17.3 ± 8.8 μM) due to the expression of the T-DNA encoded iaaH and iaaM genes and their enzyme activity (own data and Thomashow et al., 1986). Application of 1 μM IAA, a concentration found in wounded and uninfected *Arabidopsis* stems (0.8 ± 0.2 μM), stimulated growth of *Agrobacterium* cells, whereas growth stimulation vanished at 10 μM and higher IAA concentrations (personal communication, J. Ludwig-Mueller, Technical University Dresden, Germany). It is known that the effect of auxin is strongly dose dependent with a growth promoting effect at low concentrations and an inhibitory effect at high concentrations, which slightly varies dependent on the plant and tissue type. One may speculate that at initiation of infection, the relatively low auxin levels of agrobacterial cells and/or of wounded plant tissue stimulate growth of agrobacteria, whereas the higher concentrations produced in the crown gall inhibit virulence as well as growth of *Agrobacterium*. Such an antagonistic auxin effect would promote transformation of the host cell at the beginning of the infection process and inhibit agrobacterial virulence and growth to prevent further transformation events in developing crown galls. In contrast to auxin, the role of cytokinin signaling in plant susceptibility is well known. Recently, it has been shown that cytokinin secreted by *Agrobacterium* controls virulence via bacterial cell growth and vir gene expression at early stages of the infection process (Hwang et al., 2010). Some, but not all plant species showed a cytokinin-dependent increase in transformation efficiency (Hwang et al., 2013). *Agrobacterium*-derived cytokinin not only acts on bacterial physiology but also influences host gene expression via the classical cytokinin-dependent signaling pathway including cytokinin receptors and the phosphotransfer cascade (Sardesai et al., 2013). Activation of this signaling cascade through agrobacterial-derived cytokinin results in inhibition of gene expression of the *Arabidopsis* MYB family transcription factor, MTF1 (Sardesai et al., 2013). MTF1 turned out to be a negative regulator of transformation susceptibility by blocking expression of the integrin-like protein At14a, a plant membrane receptor. At14A serves as anchor points for bacterial attachment at the host cell surface. Thus, at early stages of infection agrobacterial auxin and cytokinin manipulates plant phytohormone signaling pathways to prepare the host cell for transformation.

In addition to auxin and cytokinin, plant defense signaling involves a network of interconnected pathways in which salicylic acid (SA) and jasmonic acid (JA) together with ethylene (ET) function as essential signaling molecules (Kunkel and Brooks, 2002). Exogenous application of the plant defense molecule SA to *Agrobacterium* cells inhibited expression of vir genes including tzs, bacterial growth, bacterial attachment to plant cells and virulence (Yuan et al., 2007; Anand et al., 2008). However, at initiation of infection (3 h post infection) neither SA nor JA levels nor the genes of these signaling pathways are elevated in *Agrobacterium*-infected *Arabidopsis* tissues (Lee et al., 2009). At this time point only the level of 1-amino-cyclopropane-1-carboxylic acid (ACC), an ET precursor, is increased in the presence of both virulent and disarmed *Agrobacterium* strains, but not expression of marker genes of the ET-dependent defense-signaling pathway. Inoculation of melon (*Cucumis melo*) explants with *Agrobacterium* also increases ET production (Ezura et al., 2000). ET is known to trigger plant auxin production due to increased expression of plant genes involved in auxin biosynthesis (Stepanova et al., 2005). Auxin enhances host susceptibility whereas plant ET production has a negative effect on agrobacterial virulence. Application of ACC reduces *Agrobacterium*-mediated gene transfer to melon explants whereas addition of aminoethoxyvinylglycin, an inhibitor of ACC synthase, increased it (Ezura et al., 2000). A reduction in transformation efficiency results from suppression of vir gene expression, but not *Agrobacterium* growth (Nonaka et al., 2008). The promoting effect of low auxin concentrations on agrobacterial growth and the inhibiting effect of ET on virulence illustrates that both, *Agrobacterium* and the host plant control host cell transformation. Taken together, at early stages of the infection process, cytokinin and auxin produced by *Agrobacterium* cells have a promoting effect on transformation efficiency, which is in part counteracted by the inhibitory effect of host plant-derived ET and SA on agrobacterial virulence. Thus, the correct phytohormone balance decides on the success of infection.

### HYPERSENSITIVE RESPONSE

Examination of early events in pathogenesis has demonstrated that virulent *Agrobacterium* does not induce HR in *Arabidopsis* (**Figure 1A**; Lee et al., 2009). Moreover, *Agrobacterium* is able to suppress HR induced by *Pseudomonas syringae pv. phaseolus* in plants (Robinette and Matthysse, 1990). This suppression is dependent on the activity of the *iaaH* and *iaaM* oncogenes which encode enzymes for auxin synthesis, since several *Agrobacterium* transposon mutants in the *iaa* genes failed to inhibit a HR. Likewise, transcription of several genes involved in oxidative stress signaling are only induced by the oncogenic, but not the T-DNA-depleted *Agrobacterium* strain (Lee et al., 2009). Production of H_2_O_2_ precedes HR, which is degraded via a chromosomally encoded catalase of *Agrobacterium* (Xu and Pan, 2000). H_2_O_2_ acts both as a local trigger for the programmed cell death and as a diffusible signal for the induction of cellular protectant genes in surrounding cells (Levine et al., 1994). Apart from its signaling functions, H_2_O_2_ is also involved in toughening of cell walls in the initial stages of plant defense by cross-linking of cell wall structural proteins (Bradley et al., 1992). Accumulation of H_2_O_2_ is prevented only at the early stages of agrobacterial infection, but proceeds in the course of tumor development (Lee et al., 2009).

## HOST RESPONSES TO CROWN GALL DEVELOPMENT

### MORPHOLOGICAL ADAPTATIONS

Development of crown galls is accompanied by profound changes in the gene expression profile, metabolism, and morphology. The uncontrolled synthesis of auxin and cytokinin by cells transformed with a T-DNA of tumorigenic Ti-plasmids drives tumor development, while the auxin to cytokinin ratios determine the crown gall morphology (**Figure 1B**). In the early days of studies about the molecular basis of crown gall development it was observed that mutations in the *tmr* locus encoding *ipt* cause rooty crown galls and those in the *tms* loci coding for *iaaH* and *iaaM* induce shooty phenotypes (Garfinkel et al., 1981; Akiyoshi et al., 1983; Barry et al., 1984; Buchmann et al., 1985; Black et al., 1994). A recent study on the T-DNA locus Atu6002 of strain C58 indicated that when the encoding protein C is expressed, it increases host cell sensitivity to auxin (Lacroix et al., 2013). In addition to the T-DNA-encoded genes, the expression of several host genes involved in auxin and cytokinin metabolism and signaling are expressed in crown galls (Lee et al., 2009). Cytokinin and auxin together with ET are known to be essential for growth of crown gall tumors and differentiation of cell types with different morphology and function (**Figure 1C**). Particularly, ET has been shown to be essential for the formation of vascular tissue and crown gall tumor development (Aloni et al., 1998; Wächter et al., 1999, 2003; Ullrich and Aloni, 2000). Application of the ET synthesis inhibitor aminoethoxyvinyl-glycine prevents vascularization in castor bean (*Ricinus communis*) stems and inhibits tumor growth completely (Wächter et al., 2003). When the ET-insensitive tomato (*Lycospersicon esculentum*) mutant, *never ripe*, is infected with virulent *Agrobacterium* cells it does not develop tumors despite integration and expression of the T-DNA encoded oncogenes for auxin and cytokinin biosynthesis (Aloni et al., 1998). Thus, neovascularization is a prerequisite for crown gall development.

Growth and expansion of crown gall tumors cause disruption of the epidermal cell layer and thereby loss of guard cells and an intact cuticle. Accordingly, expression of genes involved in cutin biosynthesis is downregulated (Deeken et al., 2006). As a disrupted surface area provides access for pathogens and leads to uncontrolled loss of water for the host plant, the crown gall surface has to be sealed. This is achieved by differentiating a periderm-like surface layer (Efetova et al., 2007). The polymerization of suberin monomers involves peroxidases for which H_2_O_2_ is the electron donor. Thus, H_2_O_2_ produced in crown galls functions in strengthening of cell walls rather than in induction of a HR. The stimulus for inducing suberization is drought stress-mediated ABA signaling (**Figure 1D**). Drought stress signaling seems to play a central role in crown gall development. ABA accumulates in crown galls in high amounts and transcription of a set of drought and/or ABA-inducible genes is elevated (Mistrik et al., 2000; Veselov et al., 2003; Efetova et al., 2007). ABA synthesis is triggered by ET as demonstrated by the application of various inhibitors of ET or ABA biosynthesis and the use of ET-insensitive or ABA-deficient tomato mutants (Hansen and Grossmann, 2000). Among the genes which play a role in drought stress protection of crown gall tumors is *FAD3,* encoding a fatty acid desaturase. The *fad3-2* mutant with impaired biosynthesis in α-linolenic acid (C18:3) develops much smaller crown gall tumors particularly in low but not high relative humidity (Klinkenberg et al., 2014). Elevated levels of C18:3 were found in the phospholipid fraction of *Arabidopsis* crown gall tumors and maintain membrane integrity under drought stress conditions. In addition to gene expression changes, crown galls accumulate high amounts of osmoprotectants, such as proline (Pro), gamma aminobutyric acid (GABA), and alpha-aminoadipinic acid. The retarded tumor growth in *abi* and *aba* mutant plants underlines the importance of an ABA-mediated drought stress-signaling pathway in crown gall development (Efetova et al., 2007).

### NUTRIENT TRANSLOCATION AND METABOLISM

Expression profiles of genes involved in energy metabolism, such as photosynthesis, mitochondrial electron transport, and fermentation together with physiological data revealed that *Arabidopsis* tumors produce C and N compounds heterotrophically and gain energy mainly anaerobically by alcoholic fermentation (**Figure 1E**; Deeken et al., 2006). The change from autotrophy to heterotrophy reduces the oxygen level in crown gall tumors thereby inducing expression of hypoxia-sensitive genes, such as *SAD6*. This gene encodes a stearoyl-acyl carrier protein desaturase, which belongs to a class of enzymes known to catalyze the first step in fatty acid desaturation, an oxygen-dependent process. Despite limited oxygen availability in crown galls, SAD6 provides the monounsaturated fatty acid, oleic acid, for membrane phospholipids (Klinkenberg et al., 2014). Thus, expression of SAD6 maintains fatty acid desaturation under hypoxic conditions.

Crown gall tumors primarily use organic carbon and nitrogen for growth and are therefore a strong sink for the host plant. Metabolites and minerals have to be provided by the host plant and translocated into the crown gall tumor (**Figure 1F**). The mechanisms of nutrient translocation and their accumulation have been studied on crown gall tumors by applying cytological staining, eletrophysiological, and ^14^CO_2_ tracer techniques as well as a viral movement protein (Marz and Ullrich-Eberius, 1988; Malsy et al., 1992; Pradel et al., 1999). Solutes enter the crown gall tumor via vascular tissue, which is connected to that of the host plant and consists of phloem for the transport of assimilates and xylem for water and minerals (Aloni et al., 1995; Deeken et al., 2003). Assimilates are produced by source leaves and are apoplastically and symplastically unloaded from the phloem in crown gall tumors. High apoplastic invertase activity indicated that sucrose is unloaded apoplastically (Malsy et al., 1992). After cleavage of sucrose by sucrose-degrading enzymes, hexoses can be taken up via hexose transporters into tumor cells. *Arabidopsis* crown galls show elevated expression of several genes encoding sucrose degrading enzymes and a monosaccharide transporter (Deeken et al., 2006). In addition, a high-affinity hexose transporter has been isolated from meristematic tobacco cells transformed with a tumor inducing T-DNA and was characterized as energy independent hexose uptake transporter (Verstappen et al., 1991). Application of the membrane impermeable fluorescent probe, carboxyfluorescein (CF) to source leaves and transient expression of the GFP-labeled potato virus X (PVX) coat protein (CP), exclusively exploiting plasmodesmata for distribution, demonstrated the existence of a symplastic transport pathway between the phloem and tumor cells (Pradel et al., 1999). Both reporters show extensive cell-to-cell movement in the parenchyma of crown gall tumors but not in uninfected stem tissues of different plant species ranging from symplastic (*Curcubita maxima*) to apoplastic loaders (*R. communis, Nicotiana benthamiana*). The disrupted and enlarged surface of the crown gall tumor drives water and mineral translocation into crown gall tumors since the evaporation rate of crown galls exceeds that of leaves and non-infected stems (Schurr et al., 1996; Wächter et al., 2003). The periderm-like layer of suberized cells that covers the crown gall surface provides a considerable diffusion resistance against water vapor, but it is not an impermeable barrier for water (**Figure 1D;** Kolattukudy and Dean, 1974; Vogt et al., 1983; Schreiber et al., 2005). Cations and anions are taken up into the tumor cells through the function of membrane-localized channels and transporters expressed in the crown gall (Deeken et al., 2003). Potassium channel mutants with impaired crown gall growth underline the importance of optimal nutrient supply for growth.

### DEFENSE RESPONSES

Gamma aminobutyric acid and Pro not only serve as osmoprotectants in drought-stress related processes of the host plant, but have also an impact on *Agrobacterium* virulence (Haudecoeur et al., 2009a, b). GABA produced in crown gall tumors can be taken up by *Agrobacterium* cells and causes a delay in accumulation of 3-oxo-octanoylhomoserine lactone (OC8HSL) and Ti plasmid conjugation. GABA activates the AttKLM operon of which the AttM lactonase degrades the quorum sensing signal, OC8HSL, thereby turning on quorum quenching to protect the host plant against infections with bacterial pathogens (Yuan et al., 2008). However, Pro interferes with the import of GABA and thereby prevents GABA-induced degradation of the bacterial quorum sensing signal OC8HSL. Thus, Pro antagonizes the GABA-induced degradation of OC8HSL and therefore may be used by the pathogen to by-pass the GABA-based host plant defense.

In addition to growth and developmental processes regulated by auxin and cytokinin, crown gall biology also involves pathogen defense signaling pathways. Hormones such as SA, JA, and ET are the primary signals inducing defense responses (López et al., 2008). In *Arabidopsis* crown galls the levels of SA and ET, but not JA, are elevated (**Figure 1G**). JA has no obvious impact on crown gall tumor development, as the development on *Arabidopsis* JA-insensitive mutants is wildtype-like (Lee et al., 2009). SA and ET contents together with the expression of pathogen-related marker genes of the SA- and ET-dependent signaling pathways increase with accumulation of the T-DNA-encoded iaa and ipt transcripts. Thus, auxin and/or cytokinin seem to be important for defense signaling in crown gall tumors, since the non-tumorigenic *Agrobacterium* strain which contains a disarmed pTiC58 does not induce expression of marker genes of the SA- and ET-dependent signaling pathways (Lee et al., 2009). It is known that high levels of auxin and cytokinin stimulate ET synthesis and its accumulation in crown galls (Goodman et al., 1986; Aloni et al., 1998; Johnson and Ecker, 1998; Vogel et al., 1998; Wächter et al., 1999). In contrast to ET, the classical marker genes of the SA-dependent signaling pathways are not induced most likely as a result of the high auxin content, which has been shown to inhibit SA responses to avoid the induction of SAR (Robert-Seilaniantz et al., 2011). Despite the lack of induction of SA-dependent defense signaling, *Arabidopsis* mutant plants with high SA levels strongly reduce while those with low SA levels promote tumor growth (Lee et al., 2009). Instead of inducing host defense pathways, high SA levels act directly on oncogenic agrobacteria by inhibiting vir gene expression and thereby reducing agrobacterial virulence (Yuan et al., 2007; Anand et al., 2008). Besides SA-mediated inhibition of *Agrobacterium* virulence, SA activates the AttKLM operon, just like GABA does, to down regulate quorum sensing in *Agrobacterium* (Yuan et al., 2008). Thus, activation of quorum quenching by auxin, SA, and GABA, is part of the plant defense program against *Agrobacterium* in the developing crown gall. In addition to SA, ET and IAA also inhibit the vir regulon and T-DNA transfer into plant cells (**Figure 1G**; Ezura et al., 2000; Nonaka et al., 2008). Thus, the interaction between the host plant and *Agrobacterium* is very much based on phytohormone cross talk which provides a balance between pathogen-defense by the host and crown gall development promoted by *Agrobacterium*.

## EPIGENETIC PROCESSES IN DNA INTEGRATION, ONCOGENE EXPRESSION, AND CROWN GALL DEVELOPMENT

### EPIGENETIC CHANGES ASSOCIATED WITH T-DNA INTEGRATION AND ONCOGENE EXPRESSION

Epigenetic changes that affect chromatin structure play an important role in regulating a wide range of cellular processes. Histones for example are subject to post-translational modification including acetylation, phosphorylation, methylation, and ubiquitination. These modifications may influence crown gall development on different levels, either by affecting chromatin structure and DNA integration or by influencing gene expression in the host tissue. Up-regulation of several members from the core histone gene families after *Agrobacterium* infection indicates that they are important for the transformation process (Veena et al., 2003). For example, *Arabidopsis* mutants lacking histone H2A are defective in T-DNA integration (Mysore et al., 2000). In addition, a truncated version of VIP1, an *Arabidopsis* protein proposed to interact with the T-DNA-protein-complex (T-complex), which is not able to interact with histone H2A, strongly decreases *Agrobacterium* tumorigenicity (Li et al., 2005). As this decrease is most likely due to a reduced T-DNA integration efficiency, this suggests that association of the VIP1 with the host chromatin is critical for integration of the T-DNA. One hypothesis of how epigenetic information affects DNA integration is that chromatin modifications surrounding double-strand breaks (DSBs) of the DNA can be recognized by the T-complex. The resulting chromatin-T-complex may then bring T-DNA into close proximity to DSBs and facilitate its integration by the DSB repair pathway (Magori and Citovsky, 2011). Alternatively, histones may also enhance transformation by protecting incoming DNA from nuclease digestion during the initial stages of transformation. Indeed, overexpression of several histone genes in *Arabidopsis* results in higher amounts of transferred DNA and increased transient transgene expression in transformed cells (Tenea et al., 2009). Other epigenetic modifications like DNA methylation do not correlate with the T-DNA integration pattern, suggesting that T-DNA integration occurs without regard to this type of modification (Kim et al., 2007). Concerning post-translational modifications of histones, RNA-mediated knockdown of two histone deacetylases (HDT1 and HDT2) decreases *Agrobacterium*-mediated transformation efficiency of *Arabidopsis* root segments (Crane and Gelvin, 2007). Histone deacetylation functions in chromatin compaction and transcriptional repression (Strahl and Allis, 2000). Therefore, the observed effect on transformation may either be a result of effects on chromatin structure or gene expression of plant factors involved in the integration process. Histone deacetylation may also influence DNA integration by affecting DSB repair, as several histone deacetylases are critical for the DNA repair process in yeast (Munoz-Galvan et al., 2013).

After T-DNA is integrated into the plant genome, the host plant often silences transgenes. Gene silencing can occur by two different mechanisms. Transcriptional gene silencing (TGS) is a result of promoter inactivation while post-TGS (PTGS) occurs when the promoter is active but the mRNA fails to accumulate. DNA methylation of promoter sequences is frequently associated with inactivation of transgenes (Linne et al., 1990; Matzke and Matzke, 1991; Kilby et al., 1992). Screening of a large collection of transgenic *Arabidopsis* lines with single T-DNA copies including a pNOS-*NPTII* reporter gene has shown that promoter methylation is required but not sufficient for transcriptional inactivation (Fischer et al., 2008). Silencing only occurs when the plants, challenged by the silencer transgene, also provide an RNA signal. Concerning local features of the host genome affecting gene silencing, repeats flanking the site of integration seem to promote inactivation whereas flanking genes rather attenuate it. RNA silencing is triggered only if the transcript level of a transgene surpasses a gene-specific threshold, suggesting that the inactivation is part of plant defense mechanism corresponding to excessively transcribed genes (Schubert et al., 2004).

Apart from the down-regulation of transgenes that are integrated into the plant genome along with the T-DNA, the T-DNA itself may also be subject to modification by the plant silencing machinery. The first comprehensive analysis of T-DNA methylation revealed that methylation can occur in different plant tumor lines induced by *Agrobacterium*. At least one T-DNA copy in each tumor genome remained unmethylated, thereby allowing oncogene expression and crown gall proliferation (Gelvin et al., 1983). Experiments using the demethylating agent 5-azacytidine indicates that methylation negatively correlates with gene expression in plant tumors (Hepburn et al., 1983). A more recent study on T-DNA methylation in crown gall tumors induced on *Arabidopsis* stems demonstrates that the oncogene sequences are only methylated to a very low degree (Gohlke et al., 2013). The two intergenic regions, which serve as promoters for expression of the oncogenes *iaaH*, *iaaM,* and *ipt,* are completely unmethylated in *Arabidopsis* crown galls. As the gene products of these oncogenes are essential for an increase in levels of cytokinin and auxin, they are always actively transcribed in crown gall tumors of *Arabidopsis* stems (Deeken et al., 2006). The low degree of T-DNA methylation in crown galls suggests that this is a prerequisite to maintain the expression levels of oncogenes required for tumor formation. Indeed, induction of DNA oncogene methylation by production of double-stranded RNAs is sufficient to repress oncogene transcription and prevent tumor development (Gohlke et al., 2013).

### EPIGENETIC MODIFICATIONS IN THE CROWN GALL GENOME

Analysis of *Agrobacterium*-infected inflorescence stalks allowed monitoring of gene expression in the crown gall tumor at later developmental stages and revealed massive changes in its transcriptome (Deeken et al., 2006). A large part of the *Arabidopsis* genome (about 22% of genes) was found to be expressed differentially between crown galls and mock-infected stems. Of these genes, a slightly higher percentage was found to be down-regulated in crown galls (12%) compared to up-regulated genes (10%). Distinct expression changes occur at genes pivotal for energy metabolism, such as those involved in photosynthesis, mitochondrial electron transport, and fermentation. This reflects the induced host cell changes from an auxotrophic, aerobic metabolism to a heterotrophic, transport-dependent, sugar-dependent anaerobic metabolism (see Nutrient Translocation and Metabolism).

Considering that a high percentage of the *Arabidopsis* genome is differentially regulated in crown gall tumors, transcriptional reprogramming probably occurs on several levels. For example, the transcript levels of several transcription factor families (MYB, bHLH, bZIP, AP domain) change after *Agrobacterium* infection (Ditt et al., 2006; Sardesai et al., 2013), thereby inducing a tumor-specific gene expression pattern. Gene expression may also be regulated by epigenetic mechanisms like chromatin modification or DNA methylation. Apart from modifications which play a role during T-DNA integration and silencing of oncogenes (see Epigenetic Processes in DNA Integration, Oncogene Expression and Crown Gall Development), DNA methylation of plant genes can also influence tumor growth (**Figure 1H**). Indeed, 8% of protein-coding genes are differentially methylated in crown galls compared to mock-infected stems, with an overall tendency toward being hypermethylated (Gohlke et al., 2013). Depending on the position of DNA methylation, different effects on the gene expression levels are observed. In agreement with trends observed for DNA methylation changes in *Arabidopsis* (Zhang et al., 2006), increased methylation at transcription start and end sites has a negative impact on gene expression, while the two processes are positively correlated in the transcribed region. Mapping of DNA methylation in tumors revealed hypomethylation in the upstream regions of genes as well as hypermethylation in transcribed regions. Both of these may, in turn, influence gene expression and contribute to the tumor-specific expression pattern. Not surprisingly, pathways that are associated with tumor development like genes associated with cell division, biotic stress, and redox regulation are differentially methylated. Changes in the methylation pattern also have an impact on tumor growth, as *Arabidopsis* mutants in *de novo* methylation pathways promote crown gall development. Intriguingly, callus induction, which like crown gall development is also associated with dedifferentiation of plant cells, is increased in the methyltransferase mutant *cmt3* (Berdasco et al., 2008). In addition, treatment with the methyltransferase inhibitor 5-acacytidine results in increased callus formation. Recently, the DNA methylation pattern has been extensively studied in calli from *Populus trichocarpa* and *Oryza sativa*. In *Oryza sativa* calli, hypermethylation was detected compared to wild-type plants (Stroud et al., 2013). Gene bodies are hypermethylated in *Populus trichocarpa* calli compared to explants, while promoter methylation is reduced (Vining et al., 2013). Consistent with the methylation pattern in crown galls, DNA hypermethylation seems to be a general feature of a dedifferentiated status.

An attempt to identify internal plant signals which may influence DNA methylation suggests that high levels of ABA induce DNA methylation of promoter sequences (**Figure 1H**; Gohlke et al., 2013). Therefore, this phytohormone may at least partly be responsible for the methylation pattern found in crown galls. It is tempting to speculate that ABA induces DNA methylation as a response to abiotic stresses such as drought stress acclimation due to the increased water loss in crown gall tumors (Schurr et al., 1996). Possibly, ABA signaling pathways are interconnected with methylation processes in crown galls, as has been suggested for *Physcomitrella patens* (Khraiwesh et al., 2010). In the future, it would be interesting to analyze ABA knockout mutants concerning their methylation pattern in order to map ABA-induced methylation changes in a comprehensive manner and thereby improve our understanding of the connection between the different pathways. In addition, other phytohormones would also be interesting to study regarding their influence on the DNA methylation pattern in crown galls, as they display not only increased levels of ABA, but also of cytokinin, auxin, ET, and JA (Veselov et al., 2003; Lee et al., 2009).

## SUMMARY AND OUTLOOK

At the beginning of infection, sensing of *Agrobacterium* does not induce a strong defense response of the host plant. *Agrobacterium* rather exploits defense responses to increase host susceptibility for transformation and host signaling pathways to promote bacterial growth. In crown galls, however, pathogen defense pathways are considerably activated and inhibit *Agrobacterium* virulence. Accordingly, the host plant is able to limit the number of further T-DNA transformation events and to control the growth dimension of crown galls, which represent a strong metabolic sink for the host plant. Metabolic and morphological adaptations accompany the development of crown galls and generate an import-oriented tissue. The heterotrophic metabolism together with anaerobically gain of energy requires translocation of metabolites, water and minerals from the plant into the proliferating crown gall tissue. As a basis for nutrient translocation the vascular tissue needs to differentiate and the disrupted and suberized crown gall surface provides the driving force for nutrient flow. In fact, the suberized surface minimizes water loss, but still allows enough evaporation of water. Membrane integrity is maintained under the low oxygen and elevated ROS levels in crown galls by adaptation of lipid metabolism. The transcriptional changes underlying the physiological changes are partially caused by differential DNA methylation of the crown gall genome. In conclusion, both *Agrobacterium* infection and crown gall growth are highly regulated processes, which are accompanied by pathogen defense of the host and counter-defense launched by *Agrobacterium*. This regulation takes place on different levels including epigenetic control of gene expression, changes in phytohormone content as well as metabolic and morphological adaptions.

Despite the fact that the *Agrobacterium*-plant-interaction has been studied since more than 100 years and is most likely one of the best-known pathogen-host-relationships, there are still some questions left, which one may aim to answer. In addition to the one raised about the role of phytohormones other than ABA on DNA methylation in crown gall development, another one would be about the molecular mechanisms of how *Agrobacterium* cells produce auxin and how auxin increases host susceptibility for transformation. Furthermore, the status and type of plant cell susceptible for T-DNA integration is as yet unknown. The knowledge about the cellular identity sensitive for transformation will improve our understanding of transformation recalcitrant plant species. Moreover, differentiation processes in crown galls do not follow the usual patterning, unlike the situation in plant organs where developmental patterning underlies a precise spatiotemporal expression of signals and their cognate receptors. Since the original/typical developmental program seems to be overruled, crown gall tumors provide a unique opportunity for studying the molecular and biochemical mechanisms underlying cellular de-differentiation as well as differentiation processes. Not all of the questions raised may be easy to address, as some require sophisticated techniques, which at first have to be developed and established. However, invention of new techniques will benefit the entire scientific community as they have done before when *Agrobacterium* became the biotechnological tool for generation of genetically modified plants.

## Conflict of Interest Statement

The authors declare that the research was conducted in the absence of any commercial or financial relationships that could be construed as a potential conflict of interest.
